# The effectiveness of a web 2.0 physical activity intervention in older adults – a randomised controlled trial

**DOI:** 10.1186/s12966-017-0641-5

**Published:** 2018-01-12

**Authors:** Stephanie J. Alley, Gregory S. Kolt, Mitch J. Duncan, Cristina M. Caperchione, Trevor N. Savage, Anthony J. Maeder, Richard R. Rosenkranz, Rhys Tague, Anetta K. Van Itallie, W. Kerry Mummery, Corneel Vandelanotte

**Affiliations:** 1 Physical Activity Research Group, Appleton Institute, School of Health, Medical and Applied Sciences, Central Queensland University, Rockhampton, QLD 4702 Australia; 2 School of Science and Health, Western Sydney University, Sydney, NSW 2751 Australia; 3 School of Medicine and Public Health, Priority Research Centre for Physical Activity and Nutrition, Faculty of Health and Medicine, University of Newcastle, Callaghan, NSW 2308 Australia; 4 School of Health and Exercise Science, University of British Columbia, Kelowna, BC V1V 1V7 Canada; 5 Griffith University, School of Allied Health Sciences, Gold Coast, QLD 4222 Australia; 6 School of Health Science, Flinders University, Adelaide, SA 5042 Australia; 7 Department of Food, Nutrition, Dietetics and Health, Kansas State University, Manhattan, KS 66506 USA; 8 School of Computing, Engineering and Mathematics, Western Sydney University, Sydney, NSW 2560 Australia; 9 Faculty of Physical Education and Recreation, University of Alberta, Edmonton, AB T6G 2H9 Canada

**Keywords:** Physical activity, Intervention, Internet, Online, Web 2.0, Older adults

## Abstract

**Background:**

Interactive web-based physical activity interventions using Web 2.0 features (e.g., social networking) have the potential to improve engagement and effectiveness compared to static Web 1.0 interventions. However, older adults may engage with Web 2.0 interventions differently than younger adults. The aims of this study were to determine whether an interaction between intervention (Web 2.0 and Web 1.0) and age group (<55y and ≥55y) exists for website usage and to determine whether an interaction between intervention (Web 2.0, Web 1.0 and logbook) and age group (<55y and ≥55y) exists for intervention effectiveness (changes in physical activity).

**Methods:**

As part of the WALK 2.0 trial, 504 Australian adults were randomly assigned to receive either a paper logbook (*n* = 171), a Web 1.0 (*n* = 165) or a Web 2.0 (*n* = 168) physical activity intervention. Moderate to vigorous physical activity was measured using ActiGraph monitors at baseline 3, 12 and 18 months. Website usage statistics including time on site, number of log-ins and number of step entries were also recorded. Generalised linear and intention-to-treat linear mixed models were used to test interactions between intervention and age groups (<55y and ≥55y) for website usage and moderate to vigorous physical activity changes.

**Results:**

Time on site was higher for the Web 2.0 compared to the Web 1.0 intervention from baseline to 3 months, and this difference was significantly greater in the older group (OR = 1.47, 95%CI = 1.01–2.14, *p* = .047). Participants in the Web 2.0 group increased their activity more than the logbook group at 3 months, and this difference was significantly greater in the older group (moderate to vigorous physical activity adjusted mean difference = 13.74, 95%CI = 1.08–26.40 min per day, *p* = .03). No intervention by age interactions were observed for Web 1.0 and logbook groups.

**Conclusions:**

Results partially support the use of Web 2.0 features to improve adults over 55 s’ engagement in and behaviour changes from web-based physical activity interventions.

**Trial registration:**

ACTRN ACTRN12611000157976, Registered 7 March 2011.

**Electronic supplementary material:**

The online version of this article (10.1186/s12966-017-0641-5) contains supplementary material, which is available to authorized users.

## Background

Physical activity improves physical and mental health, reduces the risk of chronic disease and improves general health and wellbeing [1]. It is estimated that individuals who are physically active have a 30% to 50% lower risk of chronic disease [2–4]. Physical activity is particularly important for older adults as chronic disease risk increases with age [5]. Physical activity also reduces the risk of falls by 17% in older adults [6] and improves symptoms in those diagnosed with depression or dementia [7, 8] which are more common in older adults [5, 9]. Despite the health benefits of physical activity, only 48% of Australian adults are meeting the physical activity guidelines for good health, and this is even lower in adults aged 55–65 (43%) and 65–75 (40%) [10]. Inactivity is contributing to the burden of Australia’s aging population on the health care system [11, 12]. Interventions are needed to promote physical activity in older adults to help them to maintain their health and prevent chronic disease and mental health problems as they age [13].

The Internet is an effective way to deliver physical activity interventions in adult populations [14–16]. Web-based interventions have time, geographical and financial advantages over face-to-face interventions. This enables programs to be delivered to large populations at low cost [17, 18] and they have also demonstrated that they can be as effective as face-to-face interventions [19]. Older adults are the fastest growing age group of Internet users, with 77% of Australian adults aged 55+ years already connected [20]. Web-based physical activity interventions are well accepted by older adults and older participants have been found to have greater increases in physical activity compared to younger participants [21]. As such, web-based interventions are potentially well suited to older adults, but this area is under-researched.

Challenges with web-based physical activity interventions in adults of all ages include low satisfaction, website usage and retention. This limits long-term behavioural outcomes, as higher intervention exposure is associated with more positive behavioural outcomes [22, 23]. Greater website interactivity has shown to improve website usage and engagement in middle age adults [24]. This may also be the case in older adults, however their Internet literacy remains lower, which may influence how they use more complex and interactive websites [25]. Therefore, it is not known if enhanced interactivity in a web-based physical activity intervention is effective at engaging older adults in terms of improved satisfaction, usability and website usage and if satisfaction, usability and website usage is higher in older adults with higher Internet literacy [26, 27].

Next generation Web 2.0 applications have potential to improve the interactivity and engagement of static Web 1.0 health websites with fixed content. Web 2.0 applications are aimed at giving users control of how information is generated and shared, and include social networking, blogs, wikis, podcasts, mash-ups and video sharing sites. Web 2.0 has become common on the Internet, and users have become accustomed to this level of interactivity [28, 29]. In response to the high use of Web 2.0 applications, some recent web-based physical activity interventions have included Web 2.0 applications [30]. Maher, Lewis [30] found in their systematic review on web-based health behaviour change interventions incorporating online social networks that 9 out of 10 interventions lead to positive health behaviour changes. Social networking use is higher and more frequent in younger adults, with 79% of adults 30–49 years being Facebook users in 2015 [29]. However, there has been an increase of older adults using such applications. For example, the percentage of older adults aged 50–65 years and 65+ years with Internet access who used the social networking site, Facebook rose between 2012 to 2015 (52 to 64% and 35 to 48% respectively) [31]. Despite the rise in the number of older adults using Web 2.0 applications, older adults’ lower and less frequent use of Web 2.0 features may mean that they have a lower satisfaction and usage of Web 2.0 features in a web-based physical activity intervention when compared to younger adults.

This study builds on a previous RCT that demonstrated greater website usage and physical activity improvements of a Web 2.0 website for increasing physical activity compared to a Web 1.0 website in a sample with high age variability [32]. The first aim of this study was to determine whether an interaction between intervention (Web 2.0 and Web 1.0) and age group (<55y and ≥55y) exists for intervention satisfaction, usability and website usage (assessed through non-usage attrition, website visits, time on site, days with step entry). The second aim of this study was to determine whether an interaction between intervention (Web 2.0, Web 1.0 and logbook) and age group (<55y and ≥55y) exists for intervention effectiveness (changes in moderate to vigorous physical activity and step counts). The third aim was to investigate whether an interaction between Internet self-efficacy and intervention group (Web 2.0, Web 1.0 and logbook) exists for satisfaction, usability, website usage, and intervention effectiveness in older adults.

## Methods

### Trial design

This paper used data from the WALK 2.0 study [32], a three-arm randomised controlled trial investigating the efficacy of a web-based physical activity intervention with Web 2.0 features in comparison to a Web 1.0 physical activity intervention and a physical activity logbook. Outcomes were assessed at baseline and at 3, 12, and 18 months. A detailed description of the trial protocol can be found elsewhere [33]. The findings demonstrated that the Web 2.0 group had higher levels of website engagement and physical activity changes compared to the Web 1.0 group at 3 months but not at 12 and 18 months [32]. The data was collected according to CONSORT guidelines (see Additional file 1 for CONSORT checklist).

### Recruitment and participants

Recruitment methods for the WALK 2.0 study have been described in detail elsewhere [34]. In summary, a total of 15,526 Australians were invited to participate in the trial. Recruitment was undertaken through personalised letters to 7000 individuals in Capricornia (Central Rockhampton, QLD) and 7000 individuals in Werriwa (South Western Sydney, NSW) whose addresses were obtained from the Australian Electoral Commission database. Emails from the Population Research Laboratory to past research participants who indicated they would be interested in participating in future research and emails delivered through University email lists were also implemented.

An eligibility survey was sent along with the recruitment letters for potential participants to complete if they were interested in participating. An online version of the survey was also available. Participants were eligible to participate if they 1) lived or worked in Rockhampton or South Western Sydney, 2) were interested in increasing their physical activity, and 3) were over 18 years of age. Participants were excluded from the project if 1) they did not have access to the Internet, 2) were unable to speak/read English, 3) were engaging in moderate-to-vigorous PA (MVPA) for 30 min on 5 or more days per week, assessed with the question “as a rule, do you do at least half an hour of moderate or vigorous exercise (such as walking or a sport) on five or more days a week?” 4) had an existing chronic medical condition potentially making them at risk of injury or ill health from increasing their physical activity (assessed using the Physical Activity Readiness Questionnaire, PAR-Q) and 5) had previously participated in the 10,000 Steps program (www.10000steps.org.au) (see Additional file 2 for more infomation on sample size, participant recruitment and how missing data were handled).

### Procedure

**Fig. 1:**
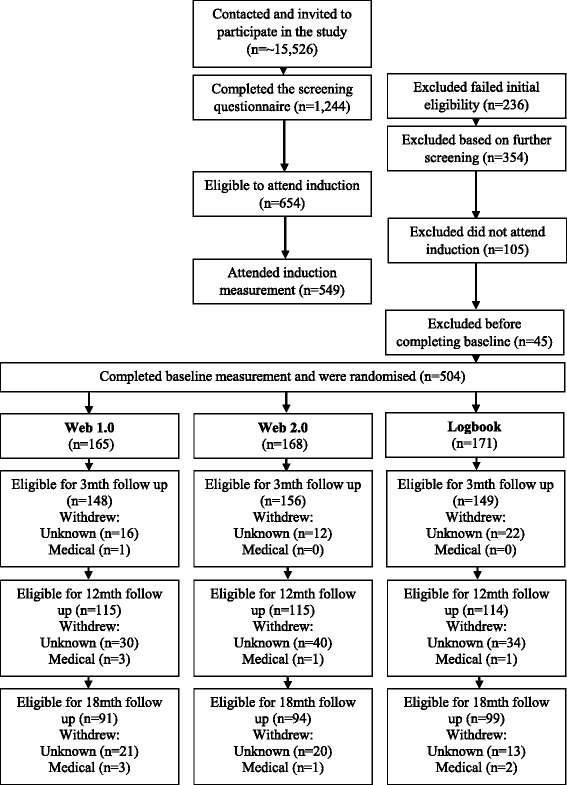
Participant flow diagram

Eligible participants were invited to attend an induction session to receive detailed information about the study and provide informed consent. Baseline physical activity data was then collected through an ActiGraph activity monitor for a week before participants attended their baseline measurement session. All measurement sessions (baseline, 3, 12 and 18 months) were conducted face-to-face to collect anthropometic measures and self-report questionnaire responses. ActiGraph monitors were posted to participants a week before they attended their 3, 12 and 18 month measurement sessions. After baseline measures were collected participants were given a pedometer to track their steps and were randomly allocated to one of the three trial arms using equal groups random allocation performed through a computer-generated algorithm (Fig. 1). A project manager enrolled and assigned participants to groups during March 2012–June 2013.

### Interventions

#### Web 1.0

Participants in the Web 1.0 group gained access to the existing 10,000 Steps website (www.10000steps.org.au). The 10,000 Steps Australia project is a community based physical activity project which has been running since 2001 and is funded by a state-wide health authority in Queensland, Australia. In conjunction with the use of a pedometer, the project specific website allows participants to keep track of the number of steps they take every day, set goals and participate in challenges. The website uses standard Web 1.0 features such as data entry and text forum submissions, based on the users’ individual, static interactions with the site. Educational materials are also available on the website. Inter-participant communication is limited to a public forum and data feed from a virtual walking buddy feature, which enables a user to share their step log with another user. Users must know the email address of their walking buddy to connect.

#### Web 2.0

Participants in the Web 2.0 group gained access to a newly developed website (www.walk.org.au). The WALK 2.0 website was developed to add to the 10,000 Steps website functionality with the aim to create a more interactive environment containing additional Web 2.0 features to increase opportunities for contact between participants. The Web 2.0 features included ‘status updates’, streams, blogs, internal emails, and forum posts. Participants had a personalised home page, allowing them to access specific information about their progress, choose features such as mapping their favourite walks using a Google ‘mashup’ tool, opportunistically ‘befriend’ other users, view their ‘friend’s’ updates, make comments and invite friends and family not part of the intervention study to join the site. Users also had a personal profile page, which allowed them to share personalised updates with their ‘friends’ on the site.

#### Logbook

Participants in the control condition were instructed not to register or use the publicly available 10,000 Steps website and received access to a paper-based logbook. The logbook allowed participants to record their steps and monitor their progress and provided participants with hard copy educational materials which were available on the intervention websites (see Additional file 3 for TIDieR checklist of the interventions).

### Measures

#### Demographics

Participant demographics were collected including age, gender (male, female), education (higher education, trade/diploma, high school), occupation (white collar, blue collar, professional, other), income (<$1000, $1000–$1999, $2000–$5000+ per week), and employment (full time, part time/casual and other). Age was categorised into (<55y and ≥55y). Although the standard cut off used to define ‘older adults’ is 65 years, the ≥55y group is a useful target for physical activity interventions to help them to establish healthy physical activity habits and reduce their risk of disease before they enter into the 65+ age group [35]. People over 55y have lower Internet literacy than younger age groups and it is therefore likely that they will interact and engage with Web 1.0 and Web 2.0 features differently to younger adults [25]. Further, the analyses for this study would be under-powered if the age cut off was higher.

#### Internet self-efficacy

Participants’ confidence in their ability to use the Internet was measured using the Internet Self-Efficacy Scale (ISES) which has a good validity and internal consistency [36]. The ISES uses 8 items assessed on a 7-point Likert scale to assess a user’s understanding of Internet hardware and software, confidence in gathering information using the Internet and learning skills to use Internet programs, and ability to troubleshoot and resolve Internet problems. The mean average of participant’s responses to the 8 items was calculated as a summary score (range 1–7).

#### Physical activity

The ActiGraph GT3X activity monitor (http://www.theActiGraph.com) was used to objectively measure minutes of MVPA and steps per day. The validity and reliability of the ActiGraph GT3X has been established [37, 38]. The activity monitor was worn for 7 full days during waking hours, except when swimming or bathing and participating in contact sports. Following past research valid wear time was set as at least 600 min wear time per day on at least 5 days within a 7-day time-period [39]. Participants were shown how to wear the ActiGraph GT3X activity monitor in the induction session. The ActiGraph GT3X was affixed to an elastic belt and worn on the waist.

#### Anthropometric measurements

Height and weight was measured by project staff to determine BMI. Height and weight was measured with the participant standing normally, with feet together and head in the Frankfurt plane, using Seca 700 mechanical balance scales and a Seca 220 measuring rod (Seca GmbH, Hamburg). Participants were asked to remove their shoes and any heavy personal items/items of clothing prior to measurement.

#### Satisfaction

Satisfaction was assessed on a 5-point Likert scale in which the Web 1.0 and Web 2.0 participants were asked to indicate how much they agreed with the following statements about their website; ‘I can easily find my way around,’ ‘I like the overall presentation,’ ‘the information is useful,’ ‘the information is easy to understand,’ ‘the information is credible,’ ‘it helped me to better monitor my physical activity,’ ‘it helped me to increase my physical activity.’ Responses were summed together to create a total satisfaction score (range 7–35).

#### Usability

Usability of each intervention was investigated at each follow-up time point using the System Usability Scale (SUS). The SUS includes 10 questions about how easy the website was to use with 5-point Likert scale responses. A summary usability score was calculated (range 0–100). The validity and reliability of the SUS is well established [40].

#### Website usage

Website usage for the Web 2.0 and Web 1.0 intervention groups was measured using Google analytics. Specifically, the frequency of step log entries, and time on website (in seconds) and number of visits to the website were recorded. The number of weeks between baseline and the first occurrence of not entering steps over a two-week period was recorded as the time for non-usage attrition to occur. These measures are commonly used to record participants’ engagement with websites [32, 41].

### Analysis

For aim 1, to test for an interaction between age group (younger, older) and intervention group (Web 2.0 and Web 1.0) for satisfaction, usability and website usage (website visits, time on site, and days with step entry) at 3, 12 and 18 months, 5 generalised linear models were calculated. A tweedie model with log link was used for each of the website usage measures due to each being negatively skewed and a linear model was used for satisfaction and usability. To test for interactions between age group (younger, older) and intervention group (Web 2.0 and Web 1.0) on non-usage attrition, a survival analysis was conducted using Cox regression. For these analyses, the Web 1.0 intervention was the reference variable for intervention group and younger adults was the reference variable for age group. Analyses were adjusted for gender, BMI, education and employment.

For aim 2, to test for an interaction between age group (younger, older) and intervention group (Web 2.0, Web 1.0 and logbook) for physical activity (MVPA and steps per day) changes over time (3, 12 and 18 months), intention-to-treat linear mixed models using maximum likelihood estimation were conducted. An analysis with logbook as the reference group was conducted to compare the Web 2.0 and Web 1.0 groups to the logbook group, and another analysis with Web 1.0 as the reference group was conducted to compare the Web 2.0 group to the Web 1.0 group. Younger adults were used as the reference variable for age group and baseline was used as the reference variable for time. Analyses were adjusted for activity monitor wear time, gender, BMI, education and employment.

For aim 3, to test for an interaction between Internet self-efficacy scores and intervention group (Web 2.0 and Web 1.0) for older adults’ satisfaction, usability and website usage (website visits, time on site, and days with step entry) at 3, 12 and 18 months, 5 generalised linear models were calculated. A tweedie model with log link was used for the website usage measures due to each being negatively skewed and a linear model was used for satisfaction and usability. To test for an interaction between Internet self-efficacy scores and intervention (Web 2.0 and Web 1.0) for older adults’ non-usage attrition, a survival analysis was conducted using Cox regression. The Web 1.0 intervention was the reference variable for intervention group and analyses were adjusted for gender, BMI, education and employment. Next, to test for an interaction between Internet self-efficacy scores and intervention group (Web 2.0 and Web 1.0) for older adults’ physical activity (MVPA minutes per day and steps per day) changes over time (baseline to 3, 12 and 18 months), intention-to-treat linear mixed models were conducted. For each outcome variable (MVPA minutes per day and steps per day), an analysis with logbook as the reference group was conducted to compare the Web 2.0 and Web 1.0 groups to the logbook group. Baseline was the reference variable for time. Activity monitor wear time, gender, BMI, education and employment were included as covariates.

## Results

### Demographics

Baseline characteristics by age group are presented in Table 1. In total, 65% of participants were female, 34% had a higher education, 46% worked full time, 32% worked as a professional and 30% had an income of over $2000AUD per week. A high percentage was obese (40%). The average age was 51 ± 13 years, the average minutes of MVPA per day was 24 ± 18 min and the average steps per day was 7248 ± 2424. Internet self-efficacy scores were 5 ± 2 out of 7.

**Table 1:** Baseline characteristics by age group

	Total (*N* = 504)	Younger (*n* = 299)	Older (*n* = 205)
	*n* (%)	*n* (%)	*n* (%)
Gender			
Male	176 (34.9)	91 (30.4)	85 (41.5)
Female	328 (65.1)	208 (69.6)	120 (58.5)
Education			
Higher	171 (33.9)	119 (39.8)	52 (25.4)
Trade/diploma	193 (38.3)	114 (38.1)	79 (38.5)
School	140 (27.8)	66 (22.1)	74 (36.1)
Employment			
Full time	234 (46.4)	168 (56.2)	66 (32.2)
Part time	111 (22.0)	73 (24.4)	38 (18.5)
Other	159 (31.5)	58 (19.4)	101 (49.3)
Occupation^a^			
Professional	159 (31.5)	116 (48.1)	43 (41.3)
White collar	102 (20.2)	70 (29.0)	32 (30.8)
Blue collar	31 (6.2)	24 (10.0)	7 (6.7)
Other	53 (10.5)	31 (12.9)	22 (21.2)
Income (AUD)^b^			
< $1000	140 (27.8)	119 (39.8)	82 (50.3)
$1000–$1999	146 (29.0)	114 (38.1)	46 (28.2)
$2000+	150 (29.8)	66 (22.1)	35 (21.5)
BMI			
Under/normal	122 (24.2)	80 (26.8)	42 (20.5)
Overweight	179 (35.5)	95 (31.8)	84 (41.0)
Obese	203 (40.3)	124 (41.5)	79 (38.5)
	M (SD)	M (SD)	M (SD)
Internet self-efficacy	5.1 (1.5)	5.6 (1.3)	4.4 (1.6)
Age	50.8 (13.1)	42.0 (8.8)	63.5 (5.4)
Daily Steps	7248 (2424)	7499 (2425)	6893 (2384)
Daily MVPA (mins)	24.0 (18.3)	26.7 (1.2)	20.1 (16.2)

^a^Missing *n* = 159, as only employed participants were asked this question ^b^Missing *n* = 68, as some participants chose not to disclose their income

### Satisfaction, usability and website usage

Descriptive statistics of satisfaction, usability and website usage and the results of the generalised linear models and Cox regression comparing these measures by age and an interaction between intervention and age are presented Table 2. Older adults were less likely to have a high satisfaction of the interventions compared to the younger participants, but they were more likely to spend more time on either website between baseline and 3 months. Older adults were also more likely to have a higher number of days with step entries across all time points and spent more time on the websites from 3 to 12 months and from 12 to 18 months. A significant interaction effect demonstrated that time spent on the website in the Web 2.0 compared to the Web 1.0 intervention from baseline to 3 months was significantly higher for older compared to younger adults.

**Table 2:** Satisfaction, usability and website usage by intervention group and age group

	Web 2.0		Web 1.0		Age Comparisons	
	Younger	Older	Younger	Older	Age group Reference = younger adults	Intervention*Age group Reference = younger adults
	M (SD)	M (SD)	M (SD)	M (SD)	HR (95% CI)	HR (95% CI)
Satisfaction (min = 1, max = 35)						
3 months (*n* = 252)	28.7 (3.7)	27.3 (3.9)	28.7 (4.2)	27.2 (3.1)	0.23 (0.06, 0.87)*	1.13 (0.17, 7.40)
12 months (*n* = 201)	26.9 (4.08)	26.8 (3.7)	27.4 (4.2)	26.4 (3.6)	0.38 (0.08, 1.71)	2.63 (0.31, 22.67)
18 months (*n* = 161)	26.8 (3.5)	25.9 (4.8)	27.2 (4.5)	26.2 (4.0)	0.32 (0.05, 1.93)	1.35 (0.10, 17.70)
Usability (System Usability Scale min = 0, max = 100)						
3 months (n = 252)	67.7 (9.7)	62.9 (10.2)	67.8 (10.4)	64.8 (8.5)	0.05 (0.00, 1.58)	0.18 (0.00, 22.65)
12 months (*n* = 200)	63.6 (9.8)	62.7 (10.7)	64.3 (10.5)	61.0 (9.5)	0.04 (0.00, 1.90)	10.51 (0.04, 2774.51)
18 months (*n* = 160)	63.1 (7.5)	60.6 (12.3)	63.0 (10.7)	61.3 (10.0)	1.19 (0.00, 15.40)	0.41 (0.00, 213.49)
Days with step entry (number/week)						
0–3 months (*n* = 332)	5.2 (2.0)	5.7 (2.1)	4.6 (2.8)	4.8 (2.3)	1.13 (0.87, 1.47)	1.09 (0.76, 1.57)
3–12 months (*n* = 297)	3.8 (2.8)	5.7 (2.2)	2.8 (3.0)	3.8 (2.9)	1.58 (1.05, 2.39)*	1.18 (0.68, 2.07)
12–18 months (*n* = 213)	2.7 (2.9)	4.5 (2.7)	1.8 (2.8)	3.2 (3.0)	1.83 (1.00, 3.35)*	1.01 (0.44, 2.30)
Time on website (seconds/week)						
0–3 months (n = 332)	596 (622)	1050 (1281)	369 (343)	485 (362)	1.34 (1.01, 1.79)*	1.47 (1.01, 2.14)*
3–12 months (n = 297)	229 (279)	618 (689)	124 (218)	212 (277)	2.23 (1.41, 3.54)**	1.53 (0.85, 2.77)
12–18 months (n = 213)	148 (293)	335 (313)	59 (97)	163 (336)	2.99 (1.55, 5.78)**	0.81 (0.34, 1.92)
Number of visits (number/week)						
0–3 months (n = 332)	3.7 (2.7)	4.6 (3.0)	1.6 (2.0)	2.1 (1.8)	1.27 (0.95, 1.69)	1.14 (0.79, 1.65)
3–12 months (n = 297)	1.9 (2.0)	3.5 (2.6)	0.8 (1.8)	1.0 (1.3)	1.53 (0.97, 2.43)	1.48 (0.83, 2.64)
12–18 months (n = 213)	1.6 (2.2)	2.6 (2.2)	0.5 (1.1)	0.7 (1.3)	1.61 (0.81, 3.22)	1.20 (0.51, 2.85)
	N (%)	N (%)	N (%)	N (%)	HR (95% CI)	HR (95% CI)
Non-usage attrition (n = 332)						
N (%) stopped using website by 18 months	87 (87.0)	47 (69.1)	86 (86.0)	53 (82.8)	0.83 (0.59, 1.16)	0.66 (0.40, 1.09)

*p < .05 **p < .003. Adjusted for gender, BMI, education and employment

### Physical activity change

**Fig. 2:**
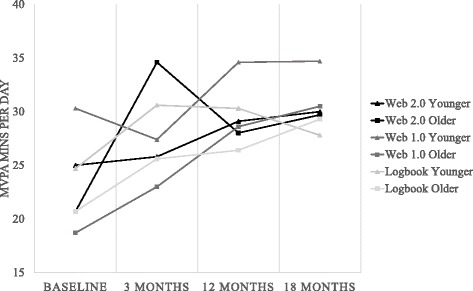
MVPA (minutes per day) by intervention, age group and time

**Fig. 3:**
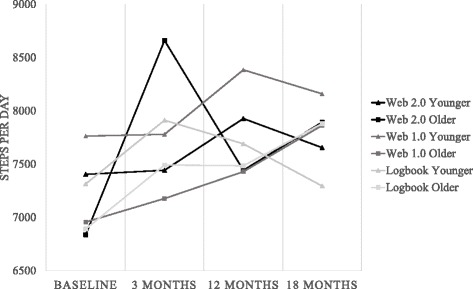
Steps (per day) by intervention, age group and time

Descriptive statistics of MVPA minutes per day and steps per day in older and younger adults in both interventions are presented in Figs. 2 and 3. Results of linear mixed model analyses comparing physical activity over time by an interaction between intervention group and age are presented in Table 3. A significant interaction between age and intervention was found for MVPA and step changes from baseline to 3 months, demonstrating that the Web 2.0 intervention was more effective than the logbook at 3 months, and this effect was significantly stronger in older compared to younger adults. No intervention group by age group interaction was seen for the Web 1.0 intervention in comparison to the logbook group, nor for the Web 2.0 intervention in comparison to the Web 1.0 intervention.

**Table 3:** Adjusted mean difference of daily MVPA minutes per day and steps per day over time by an age and intervention group interaction

		TIME*GROUP*AGE^a^		
		Web 2.0 vs Log*Older vs Younger	Web 2.0 vs Web 1.0*Older vs Younger	Web 1.0 vs Log*Older vs Younger
STEPS per day	3 months *n* = 373	1658 (70–3247)*	1354 (−274–2981)	305 (−1331–1940)
	12 months *n* = 274	−369 (−2183–1445)	−110 (−1986–1767)	−259 (−2089–1570)
	18 months *n* = 205	−367 (−2554–1820)	59 (−2146–2263)	−426 (−2581–1729)
MVPA (Mins/day)	3 months *n* = 373	13.7 (1.1–26.4)*	5.5 (−7.5–18.4)	8.3 (−4.8–21.3)
	12 months *n* = 274	2.1 (−12.7–16.9)	−2.6 (−17.9–12.7)	4.7 (−10.2–19.6)
	18 months *n* = 222	−4.6 (−21.2–11.9)	−8.5 (−25.3–8.4)	3.8 (−12.7–20.3)

*p < .05. ^a^Adjusted mean difference (95% CI) compared to baseline. Adjusted for monitor wear time, gender, BMI, education and employment

### Internet self-efficacy

A t-test revealed that older adults had a significantly lower Internet self-efficacy score out of 7 (M = 4.37, SD = 1.55) compared to younger adults (M = 5.58, SD = 1.26); *t* = (1502) 9.59, *p* < .001. The variance of Internet self-efficacy scores was also greater in older (2.41) compared to younger adults (1.58).

Within older adults, a significant interaction between older adults’ Internet self-efficacy and intervention group for time on the website was observed at 3 months (OR = 1.11, 95%CI = 1.00–1.51, *p* = .05), demonstrating that higher Internet self-efficacy was associated with more time spent on the website, and this effect was significantly stronger for the Web 2.0 intervention. There were no interactions between older adults’ Internet self-efficacy and intervention group for satisfaction, usability, non-usage attrition, days with a step entry or number of visits to the website. Within older adults, no interactions between Internet self-efficacy and intervention group (Web 2.0 and Web 1.0) were found on physical activity (MVPA or step changes) from baseline to 3 months, 12 months, or 18 months.

## Discussion

This study aimed to determine whether there were age differences in intervention satisfaction, usability, website usage and effectiveness in a Web 1.0 and Web 2.0 physical activity intervention. The original WALK 2.0 trial demonstrated higher website usage and behaviour changes in those who received the Web 2.0 intervention, and the interpretation and discussion of those outcomes has been published elsewhere [32]. However, age-related differences have not been published or discussed in relation to this study. The current study found that older adults had more website visits than younger adults, but this did not differ by intervention. Website usage (i.e., time on site) was significantly higher for the Web 2.0 intervention, and this effect was stronger in the older adult age group. This finding was unexpected as older adults in general have a lower use of Web 2.0 features such as social media [42]. It is possible that the older adults’ spent longer on the Web 2.0 intervention as they had more leisure time to interact with the Web 2.0 features compared to younger participants due to being retired. The average age for retirement in Australia is 63, which also was the average age of the older adult group [43]. The older adults may also be more willing to invest time in their health compared to younger adults as they are at an age where they are at a higher risk of developing chronic diseases [44]. The Web 2.0 features may have given them more opportunity to engage compared to the Web 1.0 website. Alternatively, it could be possible that the older adults took more time to work out how to use the Web 2.0 features, or used different Web 2.0 features than the younger participants [25]. Further research is needed to investigate how older adults interact with specific Web 2.0 features as part of a behaviour change intervention. Based on the longer time on site for older adults in the Web 2.0 intervention, future interventions targeting older adults should consider using Web 2.0 features to encourage greater website use.

No interactions between intervention and age on satisfaction, usability or non-usage attrition were observed, and older adults were less satisfied with both web-based interventions compared to younger adults at 3 months. No satisfaction differences were found at 12 and 18-months, however this may be affected by low participant numbers at these time points. It is interesting that the older adults’ satisfaction did not differ between the Web 1.0 and the more interactive Web 2.0 intervention. Therefore, whilst the Web 2.0 features were not problematic for older adults, they did not improve satisfaction. This finding suggests that the low satisfaction was not due to the features of either website, but due to the web-based method itself. Older adults may not be as comfortable as younger adults in using the Internet for behavioural interventions [45], or they may prefer face-to-face social support to help motivate them to increase their activity [46]. The lower satisfaction in older adults may also be due to the Internet use not being as well integrated into their daily lives [20] and should be considered when developing physical activity interventions for this age group.

The results of the current study demonstrated greater effectiveness of the Web 2.0 intervention compared to the logbook intervention in older compared to younger adults, whilst effectiveness of the Web 1.0 intervention in comparison to the logbook intervention did not differ by age. The increased effectiveness of the Web 2.0 intervention in older compared to younger adults is in line with past research which found that a web-based physical activity intervention providing interactive tailored advice was more effective in older compared to younger adults [21]. This finding may be due to older adults having more time to engage with interactive website components (over just reading text on a static page, which takes less time), which motivated them to increase their activity further. The older participants may have also had more time to be active compared to the younger participants [47]. Despite the effectiveness of the Web 2.0 intervention compared to the logbook intervention, particularly in older adults, there was no interaction for age and the Web 2.0 compared to the Web 1.0 intervention. Therefore we do not know if the Web 2.0 features significantly impacted behaviour change. Interactive features that provide peer or counsellor support improve engagement in behaviour change interventions across all ages [24], however further research is needed to investigate which specific Web 2.0 features are most effective at contributing to behaviour change in older adults. Furthermore, whilst our results indicate that an intervention with interactive features may be effective at improving short-term intervention effectiveness in older adults, more research is needed to investigate how such interventions can assist older adults maintain their activity levels in the long-term.

The findings revealed that Internet self-efficacy in older adults was positively associated with usability ratings and intervention satisfaction. The lower levels of satisfaction in older adults could therefore be due to this group’s lower levels of Internet self-efficacy. However, Internet self-efficacy was also positively associated with older adults’ time spent on the website; for which the effect was stronger for those in the Web 2.0 intervention. Therefore, as Web 2.0 features are more complex, they are likely to be better suited to older adults with a high Internet self-efficacy who have a greater understanding and confidence in using the Internet. Yet, the lower Internet self-efficacy in older adults was not enough to influence the overall effectiveness of the Web 2.0 intervention in this age group. These conflicting outcomes make it difficult to determine the importance of a high or low Internet self-efficacy for engagement and behaviour changes of web-based physical activity interventions for older adults, and other studies should also investigate this. On a positive note, Internet self-efficacy within older adults is likely to increase over the coming years as an increasing number of older adults have used the Internet for a significant portion of their working lives [42].

This study is the first to investigate the effectiveness of a web based physical activity intervention with interactive Web 2.0 features compared to a Web 1.0 and logbook intervention in older adults. The findings are important for informing the next generation of web-based interventions with a wide reach for older adults. Strengths of the study include the objective physical activity measures, the large sample and the long-term follow up. However due to the nature of the RCT with the long term follow up, and the quickly advancing Internet technology, the Web 2.0 technology used in the intervention may already be outdated to some extent. Further, attrition may have affected the ability to detect satisfaction, usability and website usage differences by age and intervention at 12 and 18 months and biased physical activity outcomes at 12 and 18 months. Lastly, the number of older adults over the age of 65 was small, which required an age cut-off of 55 to maintain adequate statistical power. Further research is needed to test the effectiveness of web-based physical activity interventions in older adults with a larger cohort of older adults to allow further age break down (e.g. 55–65 and 65+) and investigate the influence on other factors relevant to older adults including chronic disease status and retirement status.

## Conclusion

The findings demonstrate that web-based physical activity interventions can be more engaging and effective in older compared to younger adults, and that interventions with Web 2.0 features are particularly engaging and effective in older adults. Although the Web 2.0 intervention was not as engaging in older adults with a low Internet self-efficacy, Internet self-efficacy was not associated with older adults’ physical activity changes. Future web-based interventions targeting older adults are recommended to include Web 2.0 features to improve website usage and optimise physical activity outcomes.

## Additional files

Additional file 1: CONSORT checklist. (DOC 218 kb)

Additional file 2: Participants in Walk Trial. (DOCX 12 kb)

Additional file 3: TIDieR checklist of the interventions. (PDF 129 kb)

## Abbreviations

ISES: Internet Self-Efficacy Scale
MVPA: Moderate-to-vigorous PA
SUS: System Usability Scale
