# Supplementary material for: The effectiveness of a web 2.0 physical activity intervention in older adults – a randomised controlled trial
Source: Int J Behav Nutr Phys Act. 2018 Jan 12;15:4. doi: 10.1186/s12966-017-0641-5 (PMC5766986; doi:10.1186/s12966-017-0641-5)
Supplement: Supplementary file 2 — Participants in Walk Trial. (DOCX 12 kb) [file 12966_2017_641_MOESM2_ESM.docx]

**Participants in Walk Trial**

**Sample size**

Sample size was based on the primary outcome measure of minutes of MVPA. A review of web-based PA interventions suggests that studies which do not include aspects of Web 2.0 had a small effect on change in PA. Given the expected enhanced effect of Web 2.0, this study was powered to detect a small-to-moderate difference in MVPA between groups (Web 2.0, Web 1.0, Logbook) with 80% power at any given time point. To achieve this, ∼120 participants per group were required (α level of 0.05). The number of participants per group was inflated by 40% (n=168 per group) to account for expected participant dropout while retaining adequate power to achieve study aims at 18 months (long-term follow-up).

**Recruitment**

The primary recruitment method was personalised invitation letters sent to an extract of individuals selected randomly from the Australian Electoral Commission electoral roll, supplemented with local print media advertisements, emails to university email lists and through people registered with the university as interested in future research.

**Representativeness of sample**

The sample consisted of a higher percentage of females (65%) compared to the Australian population (50%), 72% had a post-high school education which is greater than the Australian population (59%) and 31.5% were not in paid employment which is lower than the Australian population (39%) (Australian Bureau of Statistics, 2015a, 2015b).

**How missing data were handled**

Primary analysis of endpoints was intention-to-treat, where between-group differences in physical activity changes from baseline to follow-up at 3, 12 and 18 months were assessed using linear mixed models.

**References**

Australian Bureau of Statistics. (2015a). *Education and Work, Australia, May 2015* Canberra, Australia: ABS.

Australian Bureau of Statistics. (2015b). *Population by Age and Sex, Regions of Australia, 2014* Canberra, Australia: ABS.
